# Chemometric Methods for Studying the Relationships Between Trace Elements in Laryngeal Cancer and Healthy Tissues

**DOI:** 10.1007/s12011-014-0013-9

**Published:** 2014-05-18

**Authors:** R. Dobrowolski, J. Klatka, D. Brodnjak-Voncina, A. Trojanowska, D. Myśliwiec, J. Ostrowski, M. Remer

**Affiliations:** 1 Faculty of Chemistry, Department of Analytical Chemistry and Instrumental Analysis, Maria Curie-Skłodowska University, 20-031 Lublin, Poland; 2 Department of Otolaryngology, Medical University, 20-059 Lublin, Poland; 3 Faculty of Chemistry and Chemical Engineering, University of Maribor, Smetanova 17, 2000 Maribor, Slovenia; 4 Department of Radiology, Medical University, 20-059 Lublin, Poland; 5 Faculty of Chemistry, Department of Radiochemistry and Colloid Chemistry, Maria Curie-Skłodowska University, 20-031 Lublin, Poland; 6 Analytical Department, Fertilizers Research Institute, Al. Tysiąclecia Państwa Polskiego 13A, 24-110 Puławy, Poland; 7 Department of Otolaryngology, District Hospital, 22-400 Zamość, Poland

**Keywords:** Chemometrics, Laryngeal cancer, Trace elements, ICP-OES, AAS

## Abstract

A quick and reliable method for the evaluation and classification of two types of tissues is presented. Several chemometric methods were applied to evaluate multivariate data of the tissue samples with respect to the content of trace elements. The content of Pb, Al, Zn, Cd, Cu, Ni and Co was determined in samples of healthy and cancerous tissue obtained from 26 patients. Determination was done at milligram/kilogram level with inductively coupled plasma optical emission spectrometry (ICP-OES) and atomic absorption spectroscopy (AAS) techniques. Contents of trace metals in studied tissues are not normally distributed; however, normal distribution was confirmed for log values. There is a statistically significant difference in the content of Zn, Cd, Cu and Al (*p* < 0.01) and Ni and Co (*p* < 0.05) when healthy tissue is compared to cancerous one. Correlation between contents of trace elements for studied tissues was positive; the highest was found between Zn and Cu. A chemometric methodology seems to be a promising tool for classifications of the tissue samples.

## Introduction

The human body is a very complex structure and an appropriate concentration of trace elements (the contents of which is less than 0.01 % of total body weight) is very important for its optimal operation. This is because they play an important role in numerous biochemical processes [1], e.g. zinc and copper are necessary for the proper functioning of the immune system [2, 3]. In most cases, action of those elements is based on activation and/or inhibition of certain enzymes, e.g. by changing permeability of cell membranes [4]. Although trace elements are necessary to preserve a general body homeostasis, recent studies suggest that the excessive exposure to some of them may act as pathogenic factors. Few authors have studied the relationship between a concentration of some elements in human tissue and occurrence of certain diseases, mostly cancer [1, 4–8]. Moreover, Arita and Costa [9] and Salnikov and Zhitkovich [10] have published reviews concerning genetic and epigenetic mechanisms of carcinogenesis and carcinogenic properties of some elements, to which the human body might be exposed [11–13]. Study of this topic is extremely important because of progressing industrialization of human habitations and enhanced risk of exposure.

There are two main mechanisms responsible for element epigenetics: DNA methylation and posttranslational modification of histones [9]. Currently, it is believed that cancer is a consequence of abnormal and ‘disturbed’ DNA methylation—in most cases, global methylation is lowered while locally, it may be increased [14]. Recent studies revealed that epigenetic mechanisms are connected related to many elements such as nickel [9, 15, 16], chromium [9, 10], cobalt [10, 17], cadmium [18] and copper [19]. Due to the fact that epigenetic mechanism may lead to cancer, one can postulate that concentration of the mentioned elements might be different in cancer cells as compared to the healthy ones in the same tissue.

In the presented paper, we have analysed concentrations of lead, cobalt, zinc, aluminium, cadmium, copper and nickel in laryngeal cancer and laryngeal healthy tissue. Complex statistical analysis of this data set is performed. Finally, the hypothesis claiming that concentration of analysed elements may be used as a diagnostic tool is evaluated.

## Materials and Methods

We may point out two main aims of the presented paper. Firstly, we wanted to quantify information about a single group of patients, namely, smokers diagnosed with larynx cancer. Secondly, we have compared data from two sets—concentration of trace elements in tissues classified as cancerous and in the one supposed to be healthy. The study was conducted on 26 samples obtained from squamous cell laryngeal carcinoma and 26 corresponding cancer-free laryngeal tissues from the same male patients (44 to 76 years old, average age 62) after total laryngectomy. The patients were treated in the Department of Otolaryngology at the Medical University of Lublin. Patients who were taking microelements as supplements to their diet were excluded from the study. Samples of laryngeal tissues were weighed (about 0.5 ± 0.001 g) and digested in 3 ml of Suprapur nitric acid (Merck, Germany) using the closed mineralization system Mars 5 (CEM, USA). Mineralization parameters were as follows: final temperature 195 °C, pressure 260 psi and time of mineralization 8 min. Following the mineralization, the residue was transferred to polyethylene volumetric flasks (25 ml) and filled to volume with bidistilled water. The total content of the elements was determined by inductively coupled plasma optical emission spectrometry (ICP-OES) using a sequential spectrometer, model Liberty II AX (Varian, Australia). In all cases, where concentrations were below or close to limit of detection, concentrations were determined with an atomic absorption spectroscopy (AAS) using multiple injection system of Varian SpectrAA 800 spectrometer. The concentration of elements in the studied material was calculated on a dry tissue basis. In order to determine a calculation factor, another portion of laryngeal tissues was dried to a constant mass at 105 °C using a laboratory dryer, model SML 35/250 (Zelmet, Poland); average dry mass was 27 % of the initial. The applied analytical procedure was validated using a certified reference material (CRM)—contents are traceable to a beef liver NCS ZC85050. The data obtained for CRM are in good agreement with certified values that were confirmed by the method described by Linsinger [20]. The precision of determination of all elements by the method presented can be regarded as acceptable.

Therefore, the results of determinations of trace elements content for both types of tissue were analysed statistically [21–23].

## Results

The concentrations of all analysed elements are summarized in Table 1.

**Table 1:** The concentrations of all analysed elements

	Concentration (mg/kg)	
	Healthy tissue	Cancer tissue
Pb	0.196 ± 0.068	0.119 ± 0.048
Co	0.033 ± 0.020	0.006 ± 0.007
Zn	55.2 ± 3.7	32.4 ± 3.1
Al	2.28 ± 1.02	0.55 ± 0.16
Cd	0.720 ± 0.205	0.327 ± 0.131
Cu	3.80 ± 0.84	2.30 ± 0.28
Ni	0.188 ± 0.102	0.060 ± 0.042

### Statistical Analysis

The results of all descriptors, namely, concentrations of seven elements in the two types of tissues (laryngeal carcinoma and healthy laryngeal tissue) have been investigated by different chemometric methods: the basic statistical methods for the determination of mean and median values, standard deviations, minimal and maximal values of measured variables and their mutual correlation coefficients. Statistical tests, correlation analysis, cluster analysis (CLU) principal component analysis (PCA) and linear discriminant analysis (LDA) were employed to analyse the differences between two types of tissues. All calculations and plots in the following section were done with the Statgraphics Centurion 10.0 and SPSS IBM Statistics 21. Microsoft Excel was utilized for the data preparation and generation of the result outputs.

The first test performed was the Kolmogorov-Smirnov method (implemented in software SPSS IBM Statistics 21) in order to investigate the normal distribution of the used data. This statistic quantifies distances between the empirical distribution function of the sample and the cumulative distribution function of the reference distribution or between the empirical distribution function of two samples. The test showed that concentrations were not normally distributed; therefore, transformation to logarithmic values was performed. The test showed the normal distribution for all element concentrations when they were presented as logarithmic values. All further calculations were performed with the original as well as logarithmic values of concentrations.

Next, a parametric analysis of variance (ANOVA) test was performed in order to find any differences in the content of studied elements between the two types of tissue. To be more precise—the available data were analysed in order to determine if statistically significant differences exist between the two sample groups (laryngeal carcinoma and healthy laryngeal tissues). The test showed that six out of seven descriptors, namely, Co, Zn, Al Cd, Cu and Ni were found to be significantly influenced by the two different types of tissue. Only Pb content was found not significantly different at 0.05 level of significance (see Table 2.). The lower the *p* value is, the more confident we may be about the significance of the difference between two groups. In other words, low value of *p* suggests that a content of a given element is different in a carcinoma tissue than in healthy one. Among the elements contained in different tissues, the following descriptors were found by Kruskal-Wallis test significantly influenced by the group: Zn, Al, Cd and Cu with *p* < 0.01 and Ni and Co with *p* < 0.05. A summary of investigated elements complemented by the results of ANOVA and Kruskal-Wallis test considering two groups is presented in Table 2.

**Table 2:** One way ANOVA

ANOVA						
		SoS	*df*	MS	*F*	*p* value^a^
Pb	Between groups	0.077	1	0.077	3.361	0.073
	Within groups	1.146	50	0.023		
	Total	1.223	51			
Co	Between groups	0.009	1	0.009	6.249	0.016
	Within groups	0.074	50	0.001		
	Total	0.083	51			
Zn	Between groups	6,749.692	1	6,749.692	86.782	0.000
	Within groups	3,888.884	50	77.778		
	Total	10,638.576	51			
Al	Between groups	38.960	1	38.960	11.129	0.002
	Within groups	175.044	50	3.501		
	Total	214.003	51			
Cd	Between groups	2.003	1	2.003	10.230	0.002
	Within groups	9.790	50	0.196		
	Total	11.793	51			
Cu	Between groups	28.996	1	28.996	11.198	0.002
	Within groups	129.469	50	2.589		
	Total	158.465	51			
Ni	Between groups	0.211	1	0.211	5.272	0.026
	Within groups	1.997	50	0.040		
	Total	2.208	51			

*SoS* sum of squares (variance), *df* degrees of freedom, *MS* mean square = SoS/*df*, *F* test statistics = MS(between groups)/MS(within groups), *p value* significance

^a^In chemometrics, it is common to say that difference between two groups (in this case, carcinoma/healthy tissue) is statistically significant at 95 % confidence interval if *p* value is lower than 0.05

On the base of tests that already have been described, we may postulate that tissues could be classified as healthy or cancerous based on the measured content of seven studied elements. We are aware that such a hypothesis needs to be verified with more advanced statistical and chemometric tools. Moreover, possible correlations between descriptors should be studied. Thus, we applied additional tests and analysis that are described below in detail.

The mutual correlation coefficients between all concentrations of elements were calculated. A high value of the coefficient indicates high linear correlation between contents of two elements in a tissue. The highest correlation was observed between Cu and Zn for both parametric (Pearson) and nonparametric (Spearman) tests (Tables 3 and 4, respectively). Most of the concentrations are correlated positively; however, only few of them are correlated with a factor greater than 0.3 (in italic in Tables 3 and 4). The highest value is obtained for Cu-Zn and is equal to 0.600 and 0.592 in Pearson and Spearman test, respectively, which means that contents of the elements are rather independent on each other.

**Table 3:** Pearson test

	Pb	Co	Zn	Al	Cd	Cu	Ni
Pb	1.000						
Co	0.087	1.000					
Zn	0.278	0.209	1.000				
Al	0.088	*0.319*	*0.363*	1.000			
Cd	*0.541*	−0.039	*0.390*	0.049	1.000		
Cu	0.025	0.150	*0.600*	0.106	−0.051	1.000	
Ni	0.114	*0.385*	0.257	*0.581*	0.052	0.195	1.000

Table is symmetrical. Pearson(*a*,*b*) = Pearson(*b*,*a*)

**Table 4:** SPEARMAN test

	Pb	Co	Zn	Al	Cd	Cu	Ni
Pb	1.000						
Co	0.216	1.000					
Zn	0.277	0.161	1.000				
Al	0.089	0.147	*0.370*	1.000			
Cd	*0.387*	0.072	*0.441*	0.204	1.000		
Cu	0.134	0.155	*0.592*	0.184	0.093	1.000	
Ni	0.136	0.209	0.266	0.240	0.118	0.298	1.000

Table is symmetrical. Pearson(*a*,*b*) = Pearson(*b*,*a*)

Cluster analysis (CLU) and principal component analysis (PCA) were applied for grouping tissue samples due to measured variables.

**Fig. 1:**
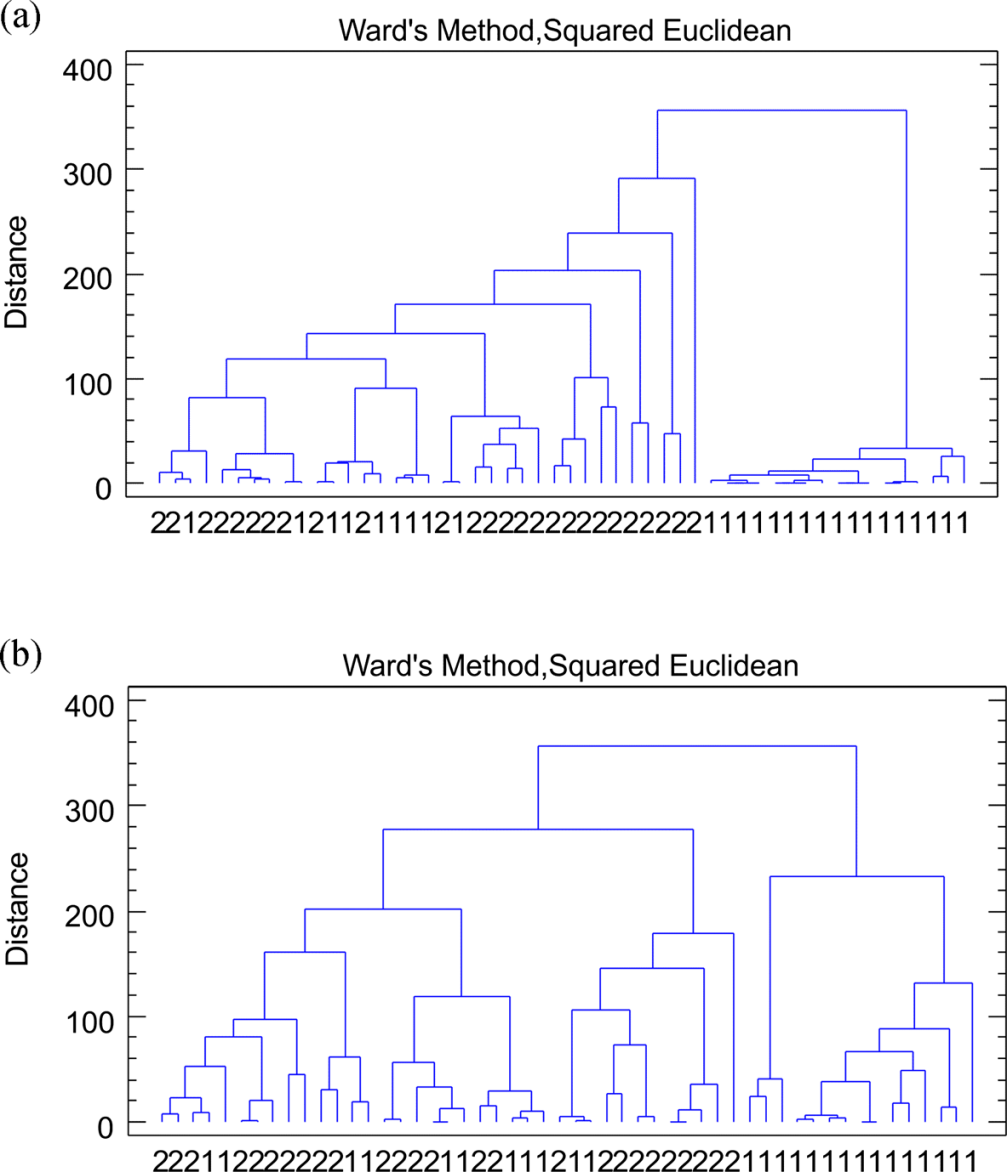
Dendrogram of the two types of tissue from 26 patients. **a** No data processing. **b** Logarithmic concentrations. Cancer-free larynx tissue (*1*) and carcinoma of the larynx tissue (*2*)

CLU is an unsupervised multivariate statistical method. The purpose of cluster analysis is to recognize groups of objects or variables based on their similarity, which is given by the distances between the objects in the multidimensional space of the chosen variables. This analysis resulted in a dendrogram shown in Fig. 1, where two groups of tissue samples are divided into two clusters, depending on the level of similarity based on Ward distance. The greater the similarity, the smaller the distance. This feature is represented by the connected branches on Fig. 1. A well-separated group is called a cluster. In this case, one on the most most-right of Fig. 1a, b is composed only by cancer-free larynx tissue samples, while cluster on the left contains mostly carcinoma of the larynx samples.

PCA is also an unsupervised method used for analysing the structure in multivariate data sets. It is used for compressing highly dimensional and correlated data into a few uncorrelated variables. The goal of PCA is to extract the important information from the data table and express this information as a set of orthogonal variables called principal components (PC). It is used for characterization of multidimensional data, providing a satisfactory representation of the studied objects by projecting the original data set from the high-dimensional space onto the lower-dimension space. Thus, PCA can transform data from multidimensional space into two dimensions without losing a considerable amount of information. With the PCA biplot, one can simultaneously demonstrate the objects (in this case, tissue samples) and the variables (contents of the elements). It is also possible to detect those variables, which are associated with the formed group of closely located objects, which means that the mutual relationships among the objects and variables can be discovered [20–22]. For the record and clarity, in this study, PCA was performed in order to obtain an overall impression about the correlation of the two types of tissues (laryngeal carcinoma and healthy laryngeal tissue) obtained from 26 patients. It was applied to the matrix composed of 52 × 7 elements. Fifty-two rows represented the tissue samples composed of seven variables. The tissue samples are characterized by the concentration of Pb, Co, Zn, Al Cd, Cu and Ni. The enumerated variables are components of the vector representation of each tissue sample that is used in further chemometric analysis. Each patient was described with two samples, one carcinoma of the larynx tissue and one cancer-free larynx tissue. Moreover, the data were additionally preprocessed before PCA. Column standardization of individual variables was performed. In this procedure, the mean of the column elements is subtracted from individual elements and divided by the column standard deviation. Consequently, each column has zero mean and unit variance.

**Fig. 2:**
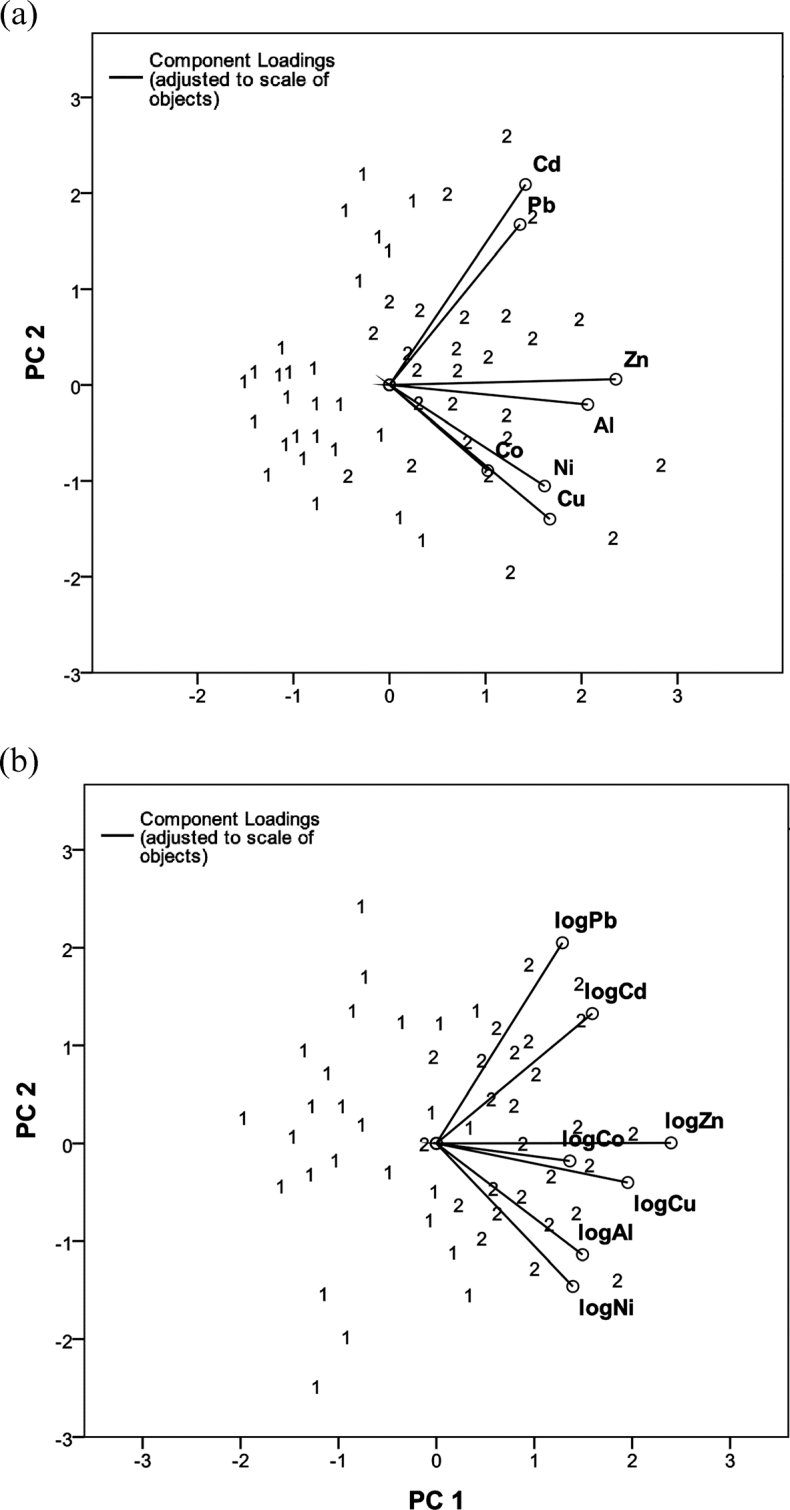
Biplot analysis of the two types of tissues. Cancer-free larynx tissue (*1*) and carcinoma of the larynx tissue (*2*). **a** No data processing. **b** Logarithmic concentrations. The closer the points to each other, the more similar tissues are (in terms of content of the studied elements)

The results showed that 52.41 % of variance was explained in the first two principal components of the transformed data. It was evident that a considerable amount of information still remains by keeping only the first two principal components. Figure 2 shows the biplot (PC1 vs. PC2) resulting from PCA of the carcinoma larynx tissues and cancer-free larynx tissues represented with seven variables. This plot displays the positions of the two types of tissue as well as all variables. It can be seen that the first principal component PC1 was mainly associated with Zn and Al concentration which are positively correlated. The second component PC2 mainly represented the descriptors Cd and Pb also showing their positive correlation. We must additionally point out that, from Fig. 2, the grouping is evident. PCA for those tissue samples, distributed within the region of larger PC1 and also the PC2 values, were samples labelled as number 2 (carcinoma of the larynx tissue). The first cluster on the left contains samples labelled as number 1 (cancer-free larynx tissue). The samples labelled with 1 are well separated from the samples labelled with 2 (carcinoma of the larynx tissue). Results are in concordance with ANOVA and Kruskal-Wallis test and show significant differences between two types of tissue.

Linear discriminant analysis (LDA) is a supervised pattern recognition method in which a classification model is constructed using the data of the objects precategorized into known categories. The calculation algorithm of LDA is trained to discriminate the objects into the given categories (classes). It is based on the determination of linear discriminants, which maximize the ratio of between-class variance and minimize the ratio of within-class variance. LDA is considered to be a dimension reduction method. For feature reduction, it is necessary to determine a smaller dimension hyper plane on which the points will be projected from the higher dimensional space. LDA selects directions, which achieves maximum separation among the given classes [22]. The calculations were performed using the software IBM SPSS Statistics 21. The validation of the LDA model was accomplished by the leave-one-out method. Altogether, 52 tissue samples were divided into two types: (1) cancer-free larynx tissue and (2) carcinoma of the larynx tissue.

**Fig. 3:**
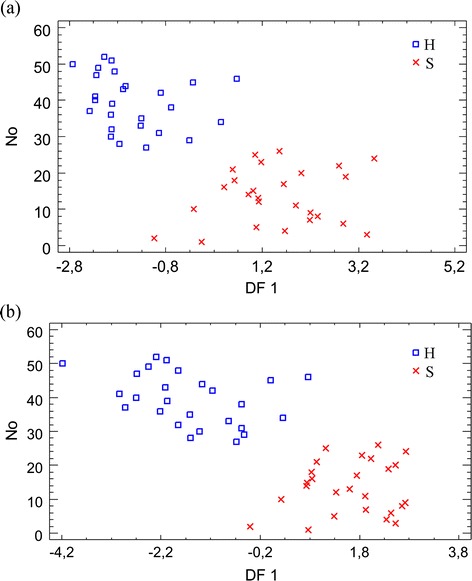
Graphical output of LDA for the 52 tissue samples that are separated in two clusters: Cancer-free larynx tissue (*1*) and carcinoma of the larynx tissue (*2*). **a** No data processing. **b** Logarithmic concentrations. The closer the points to each other, the more similar tissues are (in terms of content of the studied elements)

We found out that two well-separated clusters were evident from Fig. 3. The first cluster (1) is cancer-free larynx tissue and the second one (2) is carcinoma of the larynx tissue. Classification results for two types of tissue are presented in Tables 5 and 6. LDA classification rate was 88.5 % for the cross validation data for both original and logarithmic values of the elements’ concentration. In other words, we may say that tissue can be classified as healthy/cancerous with approximately 88 % accuracy, based only on the content of studied elements.

**Table 5:** Classification results for 52 tissue samples into predicted groups

Classification results					
			Predicted group membership		Total
			*H*	*C*	
Original	Count	*H*	24	2	26
		*C*	3	23	26
	%	*H*	92.3	7.7	100.0
		*C*	11.5	88.5	100.0
Cross-validated	Count	*H*	23	3	26
		*C*	3	23	26
	%	*H*	88.5	11.5	100.0
		*C*	11.5	88.5	100.0

*H* healthy tissue, *C* cancer tissue

**Table 6:** Classification results for 52 tissue samples into predicted groups using logarithmic concentration values of seven elements

Classification results					
			Predicted group membership		Total
			*H*	*C*	
Original	Count	*H*	23	3	26
		*C*	1	25	26
	%	*H*	88.5	11.5	100.0
		*C*	3.8	96.2	100.0
Cross-validated	Count	*H*	22	4	26
		*C*	2	24	26
	%	*H*	84.6	15.4	100.0
		*C*	7.7	92.3	100.0

*H* healthy tissue, *C* cancer tissue

We strongly believe that presented analysis allows us to state that tissue can be classified as carcinoma or cancer-free based on the concentration of elements studied in this paper.

## Conclusion

Several chemometric methods for the evaluation and classification of the two types of studied tissues were applied. These methods seem to be a very useful tool for a visualization of a multivariate data and to enable quick classifications of the tissue samples, in regard to the content of trace elements. From the statistic point of view, the contents of studied metals for samples of healthy and cancerous tissues are not normally distributed; however, normal distribution was confirmed for log values. Additionally, it was found that there is a statistically significant difference in concentrations of Zn, Cd, Cu and Al (*p* < 0.01) and Ni and Co (*p* < 0.05) when healthy tissue is compared to cancerous one; in the case of Pb, the difference is not statistically significant. Although the correlation between elements content is usually positive, the highest was found between Zn and Cu; however, neither in the Pearson test nor in the Spearman test, the value did not exceed 0.6. In most cases, tissues may be successfully classified as carcinoma or larynx free, based on the content of studied elements. We confirmed this fact by numerous statistical methods (CLU, ANOVA, LDA, PCA).

In conclusion, the statistic data confirmed that there is a difference in the trace metal levels in laryngeal cancer tissues as compared to healthy laryngeal ones. This phenomenon may be due to the changed cellular metabolism in the cancer development process. Further investigation is needed; however, if the levels of trace metals in cancerous and healthy tissues prove to be consistently different, it may provide a simple tool for differentiating both types of tissues. Most promising for use as the indicators are Al and Zn, which exhibit the greatest differences in their content between both types of tissue.
